# Human neutrophil migration and activation by BJcuL, a galactose binding lectin purified from *Bothrops jararacussu *venom

**DOI:** 10.1186/1471-2172-12-10

**Published:** 2011-01-25

**Authors:** Selene Elifio-Esposito, Luciane Tomazeli, Carolina Schwartz, Ana Paula Gimenez, Gabriel M Fugii, Luiz Claudio Fernandes, Luciana FM Zishler, Patrícia M Stuelp-Campelo, Andréa N Moreno

**Affiliations:** 1 Group for Advanced Molecular Investigation, PPGCS/CCBS, PUCPR, Curitiba, PR, Brazil; 2 Biology Course, CCBS, PUCPR, Curitiba, Brazil; 3 Pharmacy Course, CCBS, PUCPR, Curitiba, Brazil; 4 Fisiology Department, UFPR, Curitiba, Brazil

## Abstract

**Background:**

Neutrophil migration to an inflamed site constitutes the first line of the innate immune response against invading microorganisms. Given the crucial role of endogenous lectins in neutrophil mobilization and activation, lectins from exogenous sources have often been considered as putative modulators of leukocyte function. Lectins purified from snake venom have been described as galactoside ligands that induce erythrocyte agglutination and platelet aggregation. This study evaluated human neutrophil migration and activation by C-type lectin BJcuL purified from *Bothrops jararacussu *venom.

**Results:**

Utilizing fluorescence microscopy, we observed that biotinylated-BJcuL was evenly distributed on the neutrophil surface, selectively inhibited by D-galactose. Lectin was able to induce modification in the neutrophil morphology in a spherical shape for a polarized observed by optical microscopy and exposure to BJcuL in a Boyden chamber assay resulted in cell migration. After 30 minutes of incubation with BJcuL we found enhanced neutrophil functions, such as respiratory burst, zymozan phagocytosis and an increase in lissosomal volume. In addition, BJcuL delays late apoptosis neutrophils.

**Conclusion:**

These results demonstrate that BJcuL can be implicated in a wide variety of immunological functions including first-line defense against pathogens, cell trafficking and induction of the innate immune response since lectin was capable of inducing potent neutrophil activation.

## Background

Neutrophils are key players in the innate immune response and their recruitment from the microvasculature to inflammation sites includes rolling, firm adhesion, and transmigration through the vessel wall [1]. Neutrophil activation leads to directed migration with changes in cell morphology from rounded cells covered with microvilli to elongated ruffled cells [2,3]. Neutrophils exert their bactericidal activity at the inflammatory site through recognition and phagocytosis of the infectious agent, generation of toxic oxygen derivatives, and release of microbicidal molecules from their specialized lysosomes and granules [4]. This sequential process relies on neutrophil interaction with chemoattractants, cytokines and other inflammatory mediators [5].

Viperid and elapid snake venoms contain complex mixtures of pharmacologically active molecules, including enzymes and proteins without enzymatic activity, such as C-type lectins. Based on their structural and functional properties snake venom C-type lectins have been classified as true C-type lectins, which contain a carbohydrate recognition domain (CRD) and bind to a specific sugar molecule, and the C-type lectin-like domain proteins (CTLDs) with CRD-related non-carbohydrate-binding domains that do not bind to a sugar moiety [6,7], but act as factor IX/X-binding proteins and those interacting with platelet receptors [8].

*Bothrops jararacussu *venom lectin (BJcuL) is typically a C-type lectin with specificity to galactose units [9] and is unable to promote human platelets aggregation or even disrupt aggregation induced by ADP or thrombin [10]. In murine models it is capable of producing edema, increasing vascular permeability [10] and directly inducing cellular infiltration in endothelial cells of post capillary venules, creating an adhesive surface for leukocyte rolling [11]. All of these reports indicate that BJcuL may be involved in the inflammatory process and the activation of immune responses; however, these immunological properties and biological activities are still unclear. It is currently recognized that the role of neutrophils and macrophages is not restricted to phagocytosis and pathogen killing, but that these cells are essential for immunity and for building and modulating the innate response. In this study we analyzed the ability of BJcuL to induce neutrophil activation observed by cell polarization, migration, and adhesion, as well as increase in phagocytosis, generation of respiratory burst, increase of the lysosomes volume, and inhibition of spontaneous apoptosis.

## Results

### BJcuL recognizes specific glycoligands on the human neutrophil surface

**Figure 1:**
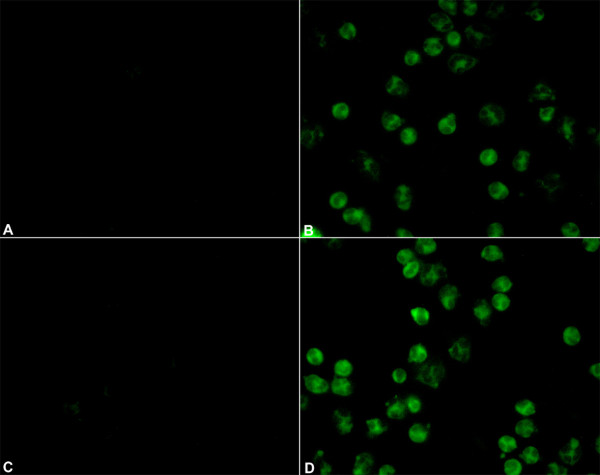
**BJcuL is evenly distributed on the surface of human neutrophils**. Isolated human neutrophils (10^6 ^cells/ml) were adhered to coverslips coated with Biobond, fixed and then incubated for 45 min at RT with (A) PBS; (B) Biotinylated BJcuL (10 μg/ml); (C) Biotinylated BJcuL pre-incubated with Gal 2 mM. (D) Biotinylated BJcuL pre-incubated with GlcNAc 2 mM. Cells were examined by fluorescence microscopy after reaction with streptavidin-FITC.

In order to localize the BJcuL glycoligands on the human neutrophil membrane, binding of the biotinylated lectin to the neutrophil was evaluated by fluorescence microscopy. Results showed that BJcuL was evenly bound on the cell surface (Figure. 1B). The interaction was completely blocked by previous incubation with the specific carbohydrate (Gal, Figure. 1C), similar to that observed in cells incubated only with PBS (Figure. 1A). Incubation with the non-specific carbohydrate (GlcNAc, Figure. 1D) did not prevent lectin binding on the cell surface.

### BJcuL induces polarization of human neutrophils

**Figure 2:**
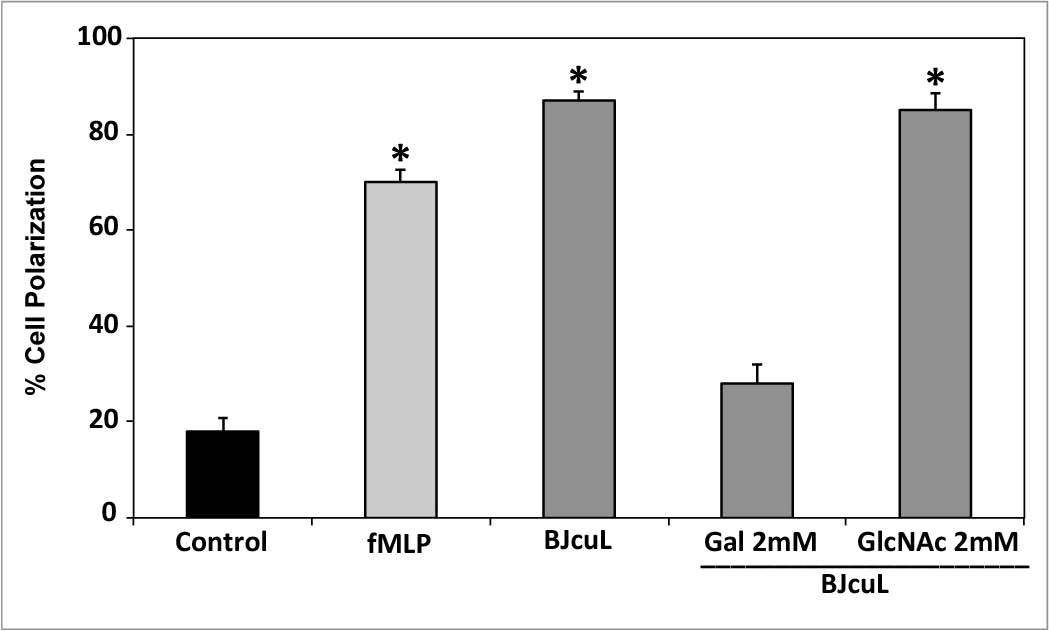
**BJcuL induces neutrophil polarization, an effect inhibited by specific sugars**. Isolated human neutrophils (10^6 ^cells/ml) were incubated with BJcuL (2.5 ng/ml) for 45 min at 37°C. In some experiments lectin was previously incubated with Gal (2 mM) or GlcNAc (2 mM). fMLP (10^-6 ^M) was used as positive controls and the RPMI was used as a negative control. Results are shown as MFI (mean ± SD from at least three independent experiments) and *p *< 0.05, Student's *t*-test.

Since neutrophils are polarized when exposed to chemoattractants, we analyzed the capacity of BJcuL to induce polarization of human neutrophils. Lectin induced 87% of the neutrophil polarization. The chemoattractant fMLP induced 70% of polarization, whereas only 18% of cells incubated with RPMI were polarized (Figure 2). BJcuL activity in inducing neutrophil polarization was reduced by 72% with Gal and was not affected by GlcNAc. These monosaccharides exerted no effect on the neutrophil polarization induced by fMLP (not shown).

### BJcuL induces neutrophil migration *in vitro*

**Figure 3:**
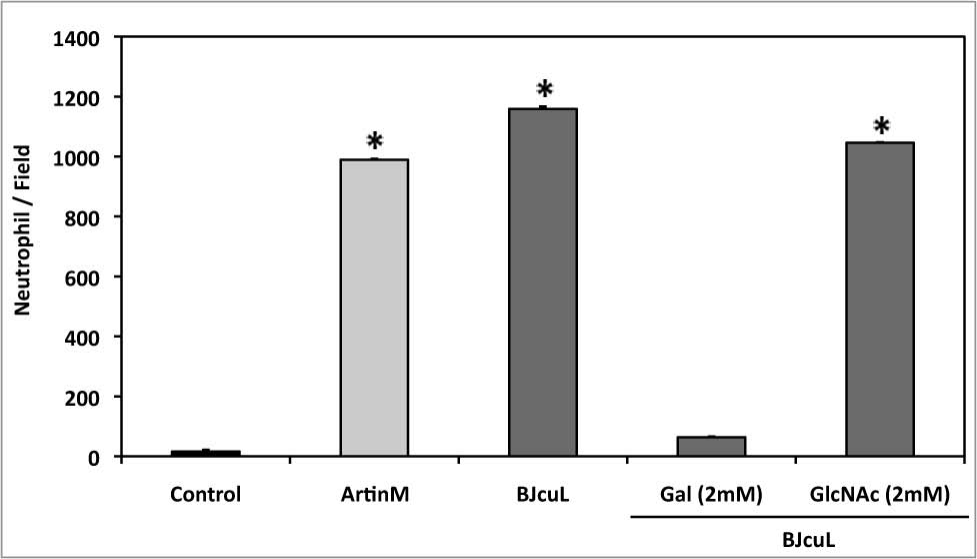
**BJcuL was able to induce neutrophil migration**. Neutrophil chemotaxis assays were carried out in 48-well Boyden microchambers. BJcuL (2.5 ng/ml), pretreated or not Gal or GlcNAc was placed in the lower chamber wells; RPMI was used as negative control. The wells of the upper chamber received isolated neutrophils (1 × 10^6 ^cells/well). The number of cells that migrated through the filter separating the two chambers was scored in each field. The figure is representative of the results of three different experiments and expressed as mean ± SD (*p *< 0.05, Student's *t*-test).

The induction of neutrophil migration by BJcuL was demonstrated *in vitro *and was dependent on sugar recognition. The original response to BJcuL (1234 ± 1.23) decreased drastically (19.34 ± 0.55) when lectin was pre-incubated with the specific carbohydrate (Gal) and was similar to that found in the absence of the stimuli (12.56 ± 0.72). This inhibition was selective since no blockage (1025 ± 1.34) was observed when BJcuL was pre-incubated with a non-specific carbohydrate (GlcNAc) (Figure 3).

### BJcuL enhances neutrophil adhesion to fibronectin and matrigel

**Figure 4:**
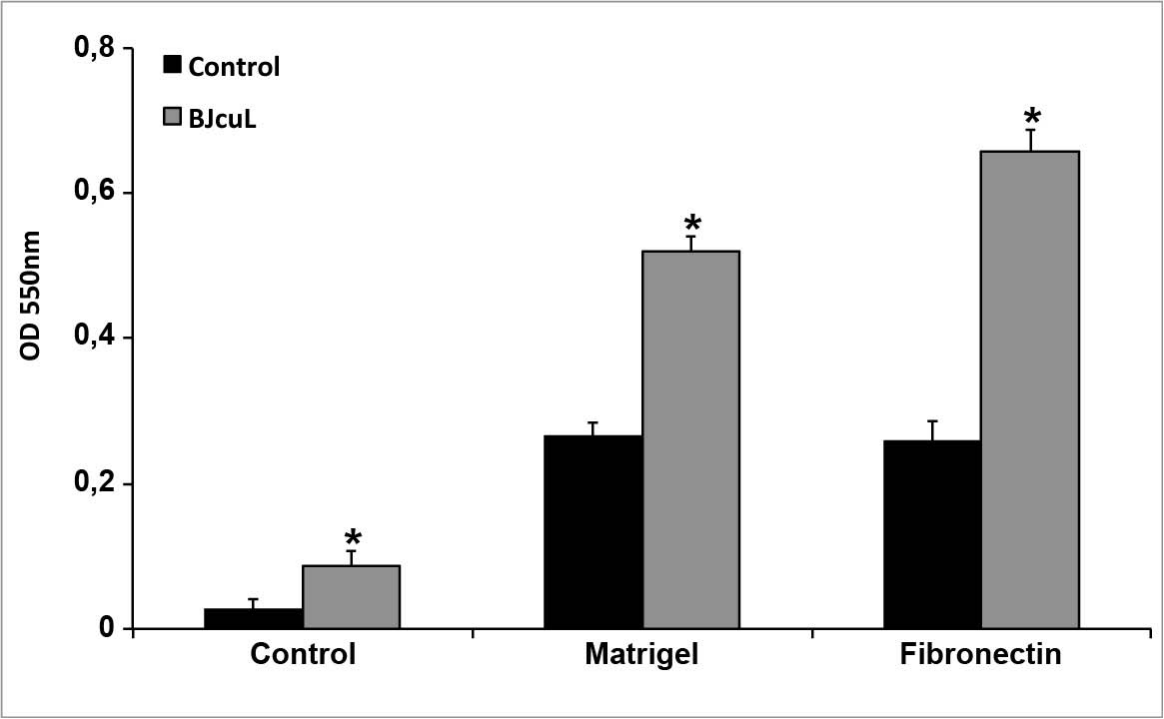
**BJcuL induces neutrophil adhesion to Matrigel and Fibronectin**. Neutrophils (1 × 10^4 ^cells/ml) were incubated with BJcuL (2.5 ng/mL) and allowed to adhere to Matrigel or fibronectin for 4 h at 37°C. The number of adhered neutrophils was determined as described in Materials and Methods. Data shown represent means ± SD of 3 experiments performed on neutrophils from different donors (*p *< 0.05, Student's *t*-test).

We previously determined that BJcuL binds to fibronectin and matrigel (data not shown) and subsequently evaluated whether or not this property was relevant for neutrophil adhesion. *In vitro *assay showed that human neutrophils spontaneously adhere to fibronectin and matrigel-coated surfaces (Figure 4). A significant increase in the adhesion was obtained when neutrophils were previously incubated with BJcuL, and was more expressive in fibronectin-coated surfaces (Figure 4).

### Increase of human neutrophil killing and phagocytosis induced by BJcuL

**Figure 5:**
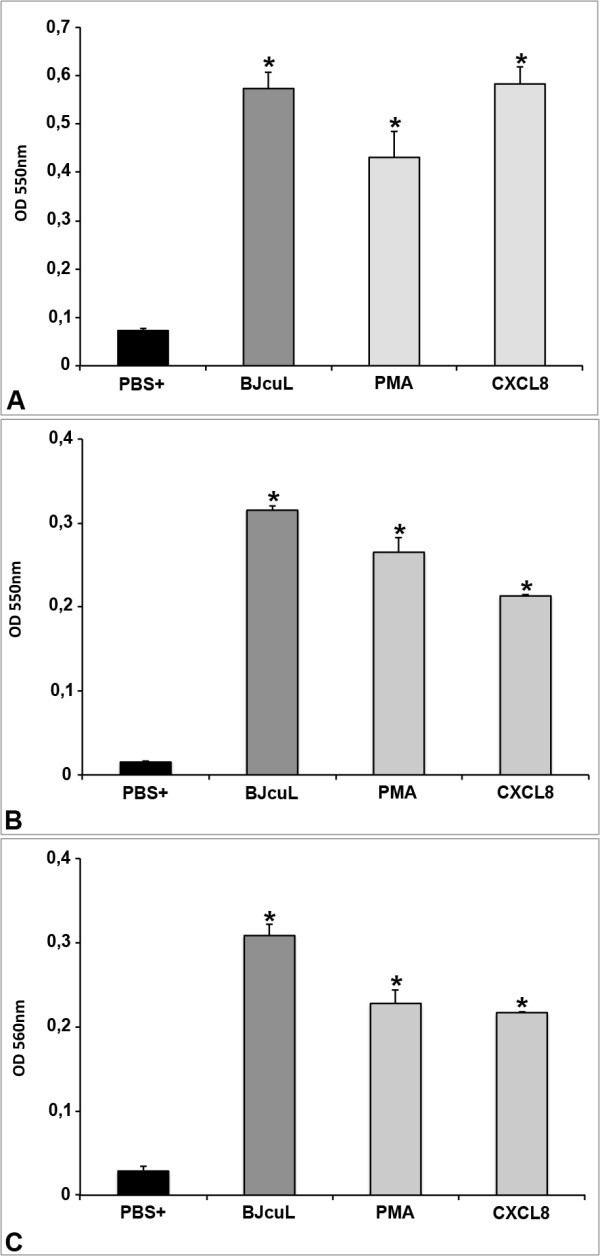
**BJcuL stimulation enhances neutrophil functions**. Following 30 min at 37°C of incubation with BJcuL (2.5 μg/ml), PMA (1 μg/ml), or CXCL-8 (2 μg/ml), neutrophil functions were evaluated. Control cells were incubated with PBS only. (A) Neutral red-stained zymozan was used as a target for the phagocytic activity of stimulated neutrophils. (B) Neutral red uptake was used to assess the volume of the neutrophil lysosomal system. (C) Superoxide production was determined by the reduction of NBT. The results are shown as mean ± SD and expressed as absorbance reading at the indicated wavelength. The values are representative of three different experiments. All stimuli provided responses that were statistically different (*p *< 0.05, Student's *t*-test) from the control.

Phagocytosis - Neutrophils were incubated with BJcuL (2.5 μg/ml) or with the positive controls PMA (1 μg/ml) and CXCL-8 (2 μg/ml) in the presence of zimosan associated with neutral red. Neutrophil phagocytosis was evaluated by subsequent measurement of absorbance. BJcuL increased neutrophil phagocytic activity, surpassing the results observed for CXCL-8 action, which has been described as a powerful inducer of phagocytosis (Figure 5A).

Uptake of neutral red by human neutrophils - Neutral red is a vital stain that accumulates in neutrophils and monocyte lysosomes [12] depending on the level of cell activation [13]. Results show that stimulated cells present increased lysosomal volume when compared to non-stimulated cells (incubated with PBS only). BJcuL (2.5 μg/ml) increased lysosomal content in neutrophils (Figure 5B), complementing the observed phagocytic activity.

Superoxide production - The superoxide anion production was also evaluated in human neutrophils after incubation with BJcuL and positive controls (as described above), and negative control (PBS). The results demonstrated that BJcuL induced superoxide release as well as its positive controls (Figure 5C).

### Anti-apoptotic effect of BJcuL on human neutrophils

**Figure 6:**
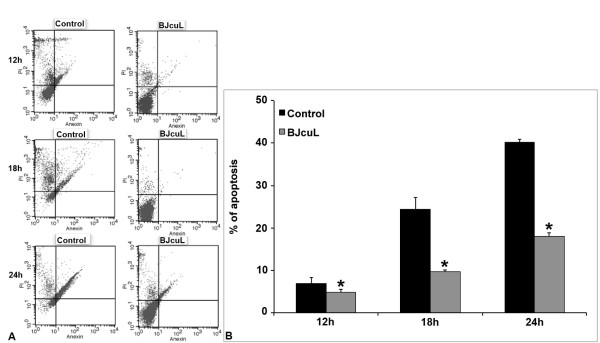
**BJcuL effects on human neutrophil spontaneous apoptosis**. Neutrophils were incubated for 12, 18 and 24 h at 37°C in the presence of BJcuL (2.5 ng/ml), labeled with Annexin-V/PI and analyzed by flow cytometry (A). Results are shown as means ± SD of the percentage of Annexin-V+/PI+ cells from three independent experiments (B). *p *< 0.05, Student's *t*-test.

Human neutrophils, incubated with BJcuL for 12, 18 and 24 hours, were labelled with Annexin-V/PI. As illustrated in Figure 6A, BJcuL was able to inhibit neutrophil late apoptosis (represented by labelled cells in the lower right quadrant) or necrosis (labelled cells in the upper quadrants), enhancing the number of viable cells (lower left quadrant). The degree of cells in spontaneous late apoptosis was 7%, 24% and 40% after 12, 18 and 24 hours, respectively, versus 5%, 9% and 17%, after incubation with BJcuL (Figure 6B).

## Discussion

Neutrophils are key cells in the innate immune system and can be activated by a variety of endogenous and exogenous substances. It has been shown that BJcuL promotes neutrophil recruitment *in vivo*, as it induces rolling and adherence of leukocytes acting directly on endothelial cells of post-capillary venules [11]. We found that BJcuL interacted with glycoligands on the neutrophil surface and that D-galactose treatment strongly abrogated lectin-cell interaction as well as cell polarization and migration. Protein-carbohydrate interactions occur in a number of processes in leukocytes [14,15] and are believed to serve as major signals for the release of pro-inflammatory mediators such as cytokines, nitric oxide, leukotrienes, and platelet activator factor [16].

*In vivo *neutrophil migration in response to an inflammatory stimulus is a crucial event mediated by direct or indirect mechanisms. The latter involve cytokines and other chemoattractants released from mast cells, macrophages and endothelial cells, after antigen recognition [17-19]. The intraperitoneal injection of BJcuL caused significant neutrophil recruitment into the peritoneal cavity of mice (data not shown) and under *in vitro *conditions BJcuL induced significant cell chemotaxis, modulating neutrophil migration by a direct mechanism that is inhibited by a specific carbohydrate. Neutrophil recruitment requires adherence to the luminal surface of vascular endothelium and thus changes the spherical cell morphology to a morphologically polarized cell that is efficient in vector migration [3]. Neutrophils stimulated by BJcuL respond promptly, presenting morphological changes that are typical of stimulated migrating cells. An intense increase in adhesion was also observed in wells coated with ECM proteins, particularly fibronectin, indicating that activation by lectin may promote the up-regulation of specific adhesion molecules, such as β-1 integrins, a family of membrane receptors devoted to cell adhesion to the extracellular matrix [20]. Integrins, in particular, have a central role in the adhesion-dependent activation for adhesion, transmigration and respiratory burst, in a rapid and reversible manner.

The capacity to stimulate oxidative burst has been previously described for other lectins that, acting directly or in concert with other mediators, could trigger or modulate neutrophil activation [21,22]. As a typical chemoattractant, BJcuL induced a rapid and expressive alteration of neutrophil ability in superoxide production and phagocytosis. We also observed that BJcuL inhibited late apoptosis in activated neutrophils until 24 hrs, but this effect seemed to be reversible once neutrophil apoptosis was gradually increased during the 24 hr period. BJcuL seemed to induce neutrophil activation and increase the life span of these cells, the time necessary for their biological function. Neutrophils undergoing apoptosis lose function and are subsequently recognized and engulfed by local macrophages, a process that is central to successful resolution of inflammation [23]. A number of factors, including cytokines, chemical mediators, and bacterial products, such as LPS, have been shown to prolong functional lifespan of neutrophils by causing cellular activation [24,25]. Stimulation of human neutrophils with TLR agonists [26] results in CXCL-8 production, up-regulation of CD11b, down-regulation of L-selectin, delayed apoptosis, enhanced phagocytosis and enhanced FMLP-induced superoxide release [27,28]. The lectin-glycoligand receptors on the neutrophil surface recognized by BJcuL are still unknown, but it is clear that this interaction induces several biological responses, thus suggesting that lectin may be a powerful pro-inflammatory molecule.

## Conclusions

In summary, the present study showed that BJcuL can recognize glycoligands on the neutrophil surface, and can promote *in vitro *polarization, migration and adhesion to extracellular matrix proteins. Lectin also induced neutrophil functional activation observed by phagocytic uptake, superoxide production, and delay in cell apoptosis. The effects observed by BJcuL seem to be mediated by cell surface D-galactose moieties. Understanding the intracellular mechanisms that induce neutrophil migration and activation by BJcuL is crucial for future investigation of therapeutic strategies.

## Methods

### Lectin Purification and Biotinylation

*B. jararacussu *lectin (BJcuL) was purified as described by Elifio-Esposito and col. (2007) [11]. BJcuL biotinylation with sulfo-NHS-LC-biotin (Pierce) was performed according to the manufacturer's recommendations.

### Human Neutrophil Isolation

Heparinized human blood from healthy volunteers was layered on a density gradient using Monopoly resolving medium (ICN Pharmaceuticals, Costa Mesa, CA, USA) and centrifuged at 400 × g for 30 min. Resulting preparations were 98% pure, and more than 95% of the neutrophils were viable as measured by trypan blue.

### Localization of the lectin glycoligands on human neutrophil surface

Neutrophils (10^6 ^cells/ml) were placed on coverslips coated with Biobond (EM Science, Fort Washington, PA, USA), fixed with 2% formaldehyde at room temperature (RT) for 20 min and incubated with biotinylated BJcuL (10 μg/ml) at 37°C for 1 hr. After washing in PBS, the cells were incubated with streptavidin-FITC (GibcoBRL) for 30 min. Cell was mounted in histological slides with Fluormount-G. In some experiments, the lectin was pre-incubated with 2 mM D-galactose (Gal) or N-acetylglucosamine (GlcNAc).

### Polarization assay

Human neutrophils were plated at 10^6 ^cells/ml and were incubated with BJcuL (2.5 μg/ml), fMLP (10^-6 ^M/well) or RPMI alone for 45 min at 37°C. In some experiments, before contact with neutrophils, the attractants were incubated for 45 min at RT with Gal or GlcNAc (2 mM). The proportion of neutrophil polarization was determined by a polarized and non-polarized cell count in a Neubauer chamber.

### Human Neutrophil migration assay

Neutrophil migration was evaluated in a 48-well Boyden microchamber (Neuroprobe, USA) by which the top and bottom wells were separated with PVP-free polycarbonate filters (5-μm pore size, Nucleopore, USA). For chemotaxis induction BJcuL (2.5 μg/well), pre-incubated or not with Gal or GlcNAc (2 mM), or RPMI alone was added to the bottom wells, whereas the neutrophil suspension (1x10^6 ^cells/ml) was placed in the top wells. Following an incubation time of 40 min, at 37°C and 5% CO_2, _the cells were stained with HEMA 3 and the number of cells migrating through polycarbonate filters was counted in five fields for each condition, assayed in triplicate.

### Adhesion assay

The adhesion assays were carried out in 96-well microplates. Each well was previously coated with fibronectin or matrigel (250 ng/ml) diluted in 0.2 M sodium carbonate pH 9,6 for 16 hrs at 4°C. The isolated neutrophils (1 × 10^4^/ml) were added in solutions of BJcuL (2.5 μg/ml) or only RPMI. After a 4 hr incubation at 37°C and 5% CO_2_, the non-adherent cells were removed by three successive washes with PBS. The adhered cell layer was fixed for 10 min with methanol, washed with PBS and then stained with 0.2% crystal violet for 5 min. After staining, the wells were repeatedly washed with PBS. The cells were lysed with sodium citrate 100 mM in ethanol and the absorbance was measured at 550 nm (Microplate Reader Benchmark - Bio-Rad Laboratories, San Francisco, CA, USA). Unspecific adhesion was evaluated in the absence of extracellular matrix protein.

### Phagocytosis assay

Neutrophil suspension (1 × 10^4^/ml) was added to the wells of a 96-well microplate and incubated with BJcuL (2.5 μg/ml), PMA (1 μg/ml) or CXCL8 (2 μg/ml) (Sigma Chemical Company) diluted in a solution containing neutral-red dye and zymosan (2.3 × 10^8 ^particles/ml) for 30 min at 37°C. After incubation, cells were fixed with Baker's formaldehyde-calcium for 30 min and then washed twice by centrifugation (453 × g for 5 min). The neutral-red stain incorporated by the cells was solubilized by acidified ethanol in each well. After 30 min, the absorbance at 550 nm was detected.

### Uptake of neutral red by activated neutrophils

The uptake of the cationic dye, neutral red, which concentrates in cell lysosomes [13], was used to assess neutrophil volume of the lysosomal system. Neutrophil suspension (1 × 10^4^/ml) was added to the wells in a 96-well microplate in the presence of BJcuL (2.5 μg/ml), PMA (1 μg/ml) or CXCL8 (2 μg/ml) and 3% neutral red in PBS for 30 min at 37°C. Cells were then washed twice with PBS by centrifugation (453 × g for 5 min). Neutral red was solubilized by 30 min incubation with acidified ethanol solution at RT and the absorbance was read at 550 nm.

### Superoxide production

Superoxide production was estimated by nitro-blue tetrazolium (NBT) reduction assay. Neutrophils (1 × 10^4 ^cells/ml) were incubated for 30 min at 37°C in the presence of BJcuL (2.5 μg/ml), PMA (1 μg/ml) or CXCL8 (2 μg/ml) and 0.1% NBT (Sigma Chemical Company). The reaction was stopped by the addition of acetic acid. The mixture was then centrifuged for 30 sec at 2500 × g. Reduction of NBT results in the formation of blue formazan, which was detected at 560 nm.

### Apoptosis assay

Neutrophils (1 × 10^6^/ml) were incubated for 12, 18 and 24 hrs at 37°C with BJcuL (2.5 μg/ml) and stained with propidium iodide and FITC-labeled Annexin-V, according to manufacturer's instructions.

### Statistical methods

All results are reported as means ± Standard Deviation (SD). Analysis of variance (ANOVA) was used to compare means between treatment and control groups. Student's t-test was used to determine if the control assay was significantly different when compared to the lectin assay. P-values of 0.05 or less were regarded as statistically significance.

## Authors' contribution

SE-E participated in the design of the study, performed the statistical analysis and drafted the manuscript. LT participated in the neutrophil adhesion, phagocytosis and superoxide production assay. CS participated in the fluorescence microscopy and polarization assay. APG carried the apoptosis assay. GMF carried the cell migration assay. LCF participated in the design of the study, mainly in the phagocytosis, volume lysosomal and superoxide production. LFMZ participated in the design of the study, mainly in the fluorescence microscopy. PMSC participated in the design of the study, specifically in the adhesion and migration assay. ANM conceived of the study, and participated in its design and coordination. All authors read and approved the final manuscript.

## References

[B1] Woodfin A Voisin MB Nourshargh S Recent developments and complexities in neutrophil transmigration Curr Opin Hematol 2010 17 9 17 10.1097/MOH.0b013e3283333930 19864945 PMC2882030

[B2] Grob PM David E Warren TC DeLeon RP Farina PR Homon CA Characterization of a receptor for human monocyte-derived neutrophil chemotactic factor/interleukin-8 J Biol Chem 1990 265 8311 8316 2186041

[B3] Sanchez-Madrid F del Pozo MA Leukocyte polarization in cell migration and immune interactions EMBO J 1999 18 501 511 10.1093/emboj/18.3.501 9927410 PMC1171143

[B4] Borregaard N Theilgaard-Monch K Cowland JB Stahle M Sorensen OE Neutrophils and keratinocytes in innate immunity--cooperative actions to provide antimicrobial defense at the right time and place J Leukoc Biol 2005 77 439 443 10.1189/jlb.0704381 15582983

[B5] Matsukawa A Hogaboam CM Lukacs NW Kunkel SL Chemokines and innate immunity Rev Immunogenet 2000 2 339 358 11256744

[B6] Zelensky AN Gready JE The C-type lectin-like domain superfamily FEBS J 2005 272 6179 6217 10.1111/j.1742-4658.2005.05031.x 16336259

[B7] Koh DC Armugam A Jeyaseelan K Snake venom components and their applications in biomedicine Cell Mol Life Sci 2006 63 3030 3041 10.1007/s00018-006-6315-0 17103111 PMC11135979

[B8] Clemetson KJ Lu Q Clemetson JM Snake C-type lectin-like proteins and platelet receptors Pathophysiol Haemost Thromb 2005 34 150 155 10.1159/000092414 16707918

[B9] de Carvalho DD Marangoni S Novello JC Primary structure characterization of Bothrops jararacussu snake venom lectin J Protein Chem 2002 21 43 50 10.1023/A:1014131115951 11902666

[B10] Panunto PC da Silva MA Linardi A Buzin MP Melo SE Mello SM Prado-Franceschi J Hyslop S Biological activities of a lectin from Bothrops jararacussu snake venom Toxicon 2006 47 21 31 10.1016/j.toxicon.2005.08.012 16309723

[B11] Elifio-Esposito SL Hess PL Moreno AN Lopes-Ferreira M Ricart CAO Souza MV Hasselmann-Zielinski F Becker JA Pereira LF A C-Type Lectin from the Venom of Bothrops jararacussu can adhere to extracellular matrix proteins and induce the rolling of leukocytes J Venom Anim Toxins Incl Trop Dis 2007 13 782 799 10.1590/S1678-91992007000400009

[B12] Sipka S Antal-Szalmas P Szollosi I Csipo I Lakos G Szegedi G Ceramide stimulates the uptake of neutral red in human neutrophils, monocytes, and lymphocytes Ann Hematol 2000 79 83 85 10.1007/s002770050015 10741920

[B13] Antal P Sipka S Suranyi P Csipo I Seres T Marodi L Szegedi G Flow cytometric assay of phagocytic activity of human neutrophils and monocytes in whole blood by neutral red uptake Ann Hematol 1995 70 259 265 10.1007/BF01784045 7599287

[B14] Kilpatrick L Johnson JL Nickbarg EB Wang ZM Clifford TF Banach M Cooperman BS Douglas SD Rubin H Inhibition of human neutrophil superoxide generation by alpha 1-antichymotrypsin J Immunol 1991 146 2388 2393 1848582

[B15] Lis H Sharon N Lectins as molecules and as tools Annu Rev Biochem 1986 55 35 67 10.1146/annurev.bi.55.070186.000343 3527046

[B16] Benjamin D Cytokine research as one of the exciting fields in immunology Methods 1997 11 81 82 10.1006/meth.1996.0390 8990092

[B17] Reddy RC Standiford TJ Effects of sepsis on neutrophil chemotaxis Curr Opin Hematol 2010 17 18 24 10.1097/MOH.0b013e32833338f3 19864946

[B18] Dias-Baruffi M Roque-Barreira MC Cunha FQ Ferreira SH Biological characterization of purified macrophage-derived neutrophil chemotactic factor Mediators Inflamm 1995 4 263 269 10.1155/S0962935195000421 18475649 PMC2365641

[B19] Moreno AN Jamur MC Oliver C Roque-Barreira MC Mast cell degranulation induced by lectins: effect on neutrophil recruitment Int Arch Allergy Immunol 2003 132 221 230 10.1159/000074303 14646383

[B20] Mambole A Bigot S Baruch D Lesavre P Halbwachs-Mecarelli L Human neutrophil integrin a9B1: up-regulation by cell activation and synergy with B2 integrins during adhesion to endothelium under flow Journal of Leukocyte Biology 2010 88 1 7 10.1189/jlb.1009704 20435742

[B21] Almkvist J Dahlgren C Leffler H Karlsson A Activation of the neutrophil nicotinamide adenine dinucleotide phosphate oxidase by galectin-1 J Immunol 2002 168 4034 4041 11937561 10.4049/jimmunol.168.8.4034

[B22] Timoshenko AV Andre S Kayser K Gabius HJ Lectin (WGA)-dependent superoxide anion release by neutrophils as a prognostic factor in lung cancer Anticancer Res 2001 21 3453 3456 11848508

[B23] Fox S Leitch AE Duffin R Haslett C Rossi AG Neutrophil apoptosis: relevance to the innate immune response and inflammatory disease J Innate Immun 2010 2 216 227 10.1159/000284367 20375550 PMC2956014

[B24] Medan D Wang L Yang X Dokka S Castranova V Rojanasakul Y Induction of neutrophil apoptosis and secondary necrosis during endotoxin-induced pulmonary inflammation in mice J Cell Physiol 2002 191 320 326 10.1002/jcp.10105 12012327

[B25] Simon HU Neutrophil apoptosis pathways and their modifications in inflammation Immunol Rev 2003 193 101 110 10.1034/j.1600-065X.2003.00038.x 12752675

[B26] Hayashi F Means TK Luster AD Toll-like receptors stimulate human neutrophil function Blood 2003 102 2660 2669 10.1182/blood-2003-04-1078 12829592

[B27] Lotz S Aga E Wilde I van Zandbergen G Hartung T Solbach W Laskay T Highly purified lipoteichoic acid activates neutrophil granulocytes and delays their spontaneous apoptosis via CD14 and TLR2 J Leukoc Biol 2004 75 467 477 10.1189/jlb.0803360 14673018

[B28] Parker LC Whyte MK Dower SK Sabroe I The expression and roles of Toll-like receptors in the biology of the human neutrophil J Leukoc Biol 2005 77 886 892 10.1189/jlb.1104636 15728244

